# Application of a Novel Long-Gauge Fiber Bragg Grating Sensor for Corrosion Detection via a Two-level Strategy

**DOI:** 10.3390/s19040954

**Published:** 2019-02-23

**Authors:** Yuyao Cheng, Chengyang Zhao, Jian Zhang, Zhishen Wu

**Affiliations:** 1 School of Civil Engineering, Southeast University, Nanjing 210096, China; chengyy@seu.edu.cn (Y.C.); 220171151@seu.edu.cn (C.Z.); zswu@seu.edu.cn (Z.W); 2 Jiangsu Key Laboratory of Engineering Mechanics, Southeast University, Nanjing 210096, China; 3 International Institute for Urban System Engineering, Southeast University, Nanjing 210096, China

**Keywords:** long-gauge fiber optic sensor, corrosion detection, strain flexibility, impact test

## Abstract

Corrosion of main steel reinforcement is one of the most significant causes of structural deterioration and durability reduction. This research proposes a two-level detection strategy to locate and quantify corrosion damage via a new kind of long-gauge fiber Bragg grating (FBG) sensor. Compared with the traditional point strain gauges, this new sensor has been developed for both local and global structural monitoring by measuring the averaged strain within a long gauge length. Based on the dynamic macrostrain responses of FBG sensors, the strain flexibility of structures are identified for corrosion locating (Level 1), and then the corrosion is quantified (Level 2) in terms of reduction of sectional stiffness of reinforcement through the sensitivity analysis of strain flexibility. The two-level strategy has the merit of reducing the number of unknown structural parameters through corrosion damage location (Level 1), which guarantees that the corrosion quantification (Level 2) can be performed efficiently in a reduced domain. Both numerical and experimental examples have been studied to reveal the ability of distributed long-gauge FBG sensors for corrosion localization and quantification.

## 1. Introduction

With the development of society, complex infrastructures such as high-rise buildings and long-span bridges have been widely constructed. During the service life of steel used in these infrastructures, steel corrosion has been considered as one of the main reasons for structural damage and deterioration, especially for those exposed to aggressive environments [1]. Over the past several years, much effort has been devoted to developing a reliable and efficient corrosion monitoring apparatus [1,2,3,4]. Zhang et al. designed an innovative Hall-effect magnetic sensor to quantify the corrosion rate for reinforced concrete structures [5]. Sunny et al. utilized a low frequency (LF) RFID sensing system to measure corrosion of steel samples in marine atmosphere and selective transient features are extracted for corrosion characterization [6]. Many conventional methods of corrosion identification, such as sensors based on macrocell measurements, sensors based on in-depth resistivity measurements, and detection based on ultrasonic techniques, have been illustrated in detail in [7]. These techniques are sensitive to structural corrosion, but fail to detect the corrosion unless the apparatus covers the corroded region. Recently, much attention has been paid to the application of fiber optical sensing techniques for corrosion detection [8,9,10,11,12,13,14,15]. As the optical fiber is small and lightweight, it can be easily attached to the surface of concrete or mounted on the steel reinforcement. With high precision and stable sensing capacity, the fiber Bragg grating (FBG)-based strain sensor is the most popular. In some studies, the main approach used to indicate the levels of corrosion is to measure the expansion in the bar diameter due to corrosion deposits [12]; this is accomplished by winding fiber optical strain sensors around the steel reinforcement in the corroded area.

However, the challenging problems described below hinder the development of strain-based corrosion detection approaches. (1) The traditional point strain sensors are unsuitable for large-scale civil engineering because they cannot successfully detect unforeseen corrosion with a short gauge length (around 1–2 cm), unless the sensors are installed in the corrosion domain [16]. Also, as a point sensor, corrosion rust can affect the fixation point [17]. (2) As a kind of structural damage, it has been found that the level of steel corrosion at the early stages of corrosion can be indicated by using a measure of the stiffness [18]. A vibration test, an effective and convenient way to excite the structure, has been widely used to extract the structural modal properties to detect damage caused by stiffness reduction. However, vibration-based corrosion detection methods are seldom studied because corrosion involves a deeper-level damage detection problem: damage quantification. Usually, civil structures with a large scale and complex form include a significant quantity of unknown parameters which will lead to slow convergence and non-uniqueness in inverse and optimal analyses [19]. Most damage detection methods may only have the capacity to locate damage and may fail to quantify the severity. Consequently, the application of strain measurements for effective structural corrosion detection and safety evaluation will be greatly enhanced if these two problems are solved.

Corresponding to the two challenging difficulties discussed above, an effective methodology for structural corrosion detection using a novel strain sensor is proposed in this paper, and two aspects have been included. (1) A kind of novel long-gauge fiber optic sensor has been developed, that can integrate both local and global information by measuring the average strains within a long gauge length (e.g., 1–2 m). Compared with the traditional strain gauge used only for “local” measurements, long-gauge FBG sensors can capture integrated information that covers the entire structure and can thus detect unforeseen damage. (2) The localization and quantification of structural corrosion damage are two different levels of corrosion detection, and they can be considered separately, and the localization and quantification can be conducted step by step. In Level 1, corrosion damage is located according to a damage index with a space resolution of the gauge lengths of the fiber optic sensors. Comprehensive development of damage assessment methodologies has been widely studied based on structural dynamic properties, such as natural frequencies, mode shapes, and their derivatives [20,21,22]. It well-known that modal flexibility is a more sensitive diagnostic indicator than mode shape and natural frequency [22,23]. In addition, strain flexibility, defined as the strain response of a structure’s element to the unit input force, has a direct relationship with structural stiffness and is much more useful for structural safety evaluation [24]. Consequently, strain flexibility is adopted here to construct a damage index. After locating the corrosion damage by a strain flexibility-based index, the quantification of Level 2 corrosion can only be conducted in the detected domain, where the number of structural parameters to be identified is less than the entire structure. Thus, it is helpful for the structural equation, solving the quantification at Level 2.

In this article, after a brief description of long-gauge FBG sensors, a step-by-step corrosion damage detection strategy is illustrated to locate and quantify corrosion; meanwhile, a solid theoretical basis is developed to guarantee accurate detection. Finally, both numerical examples and experimental tests are conducted to verify the robustness of the novel long-gauge FBG sensors and the effectiveness of the proposed method for corrosion detection in in-service structures.

## 2. Long-Gauge Fiber Optic Sensor

**Figure 1:**
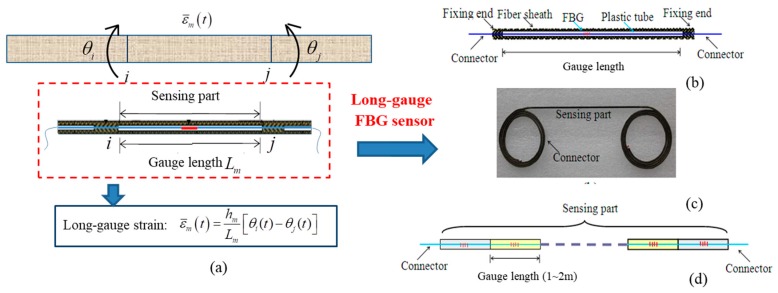
Fiber Bragg grating (FBG) sensor. (**a**) Principle of macrostrain; (**b**) Design of long-gauge FBG sensor; (**c**) Actual sensor; (**d**) Sensor arrays.

A number of sensors are utilized to measure structural responses during structural performance evaluation. These sensors are of two types: global sensing technology and local sensing technology. The former, including accelerometers and GPS, usually reflects overall structural information and is not a good candidate for detecting structural local damage. The latter, such as strain sensors and corrosion sensors, are sensitive to structural local damage, but they fail to detect local damage unless the sensor covers the damaged region. To overcome the limitation that sensors can only reflect “local” or “global” behavior (but not both), a novel long-gauge FBG sensor, shown in Figure 1, is developed to monitor structure by measuring the average strain within a long gauge length (e.g., 1~2 m), in which both local and global structural information are integrated. The principle of the long-gauge macrostrain is introduced in Figure 1a.

The designed FBG sensor with a long gauge length is illustrated in Figure 1b. One significant feature for the designed sensor is the utilization of the outer tube, which makes the in-tube fiber move freely and has the same mechanical behavior as the structure. Thus, when the FBG sensor is mounted on the structure and its two ends fixed, the strain transferred from the shift of Bragg center wavelength represents the average strain over the region the sensor covers. However, since optical fibers are fragile, bare optical fibers cannot be embedded directly into concrete. Therefore, a packaging technique is needed to protect the FBG sensor from some hostile environments, such as high temperatures, corrosion, and humidity. To enhance the measuring sensitivity, composite materials are utilized to package the optical fiber; to ensure the accurate measurements for compressive strain, the fiber is pre-tensioned before packaging to produce an initial pre-tensioned strain. The packaged long-gauge FBG sensor is shown in Figure 1c. Moreover, the long-gauge sensors can be connected in a series to make an FBG sensor array (Figure 1d) for distributed sensing. The above features provide the developed sensor with the advantage of measuring both local and global information about the structure. Therefore, a new set of strain modal identification theory can be developed, and corresponding methods will be studied for damage detection and performance evaluation for structures; this article investigates a novel method for detecting structural corrosion and quantifying the severity by processing monitoring data recorded by FBG sensors in modal space.

## 3. Two-Level Corrosion Detection Strategy Based on FBG Sensors

Long-gauge fiber optic strain sensors offer an excellent opportunity for developing a macrostrain modal identification theory and accomplishing corrosion detection by vibration test. Based on the macrostrain response measured by long-gauge FBG sensors, the theory of macrostrain flexibility identification is investigated. As the identified strain flexibility directly relates to the structural stiffness, it has clear engineering application potential for the detection of corrosion damage and further structural long-term performance evaluation.

### 3.1. Framework of the Proposed Method

**Figure 2:**
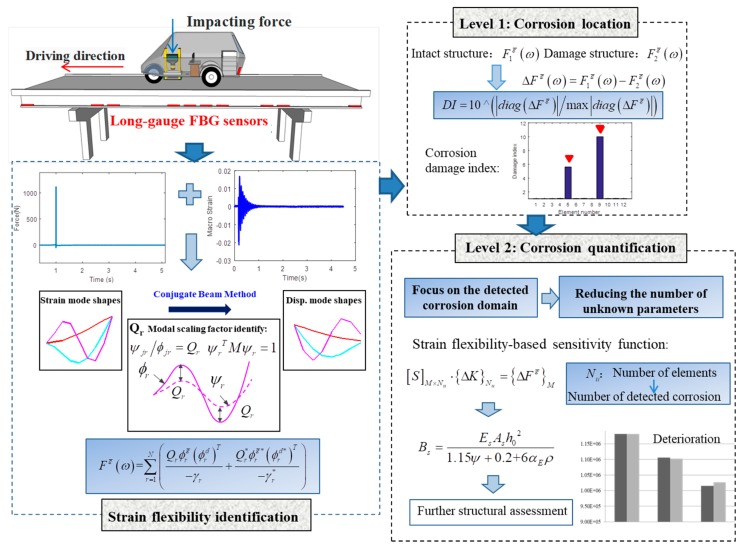
Framework of the proposed method.

The proposed two-level corrosion detection method using strain flexibility is illustrated in Figure 2. An impact test is performed on the intact structure. Based on the impacting force and dynamic macrostrain measurements, the strain frequency response function (FRF), strain modal parameter, and scaling factor will be estimated to calculate the macrostrain flexibility in the condition in which the mass is unknown. Other than the undamaged structure, the dynamic macrostrain data for a damaged structure via an impact test is also processed for structural modal identification. Based on these two strain flexibility measures, a flexibility-based damage index can be obtained by extracting the diagonal element of the flexibility difference. Then, the corrosion damage can be located for Level 1 with a space resolution of the gauge lengths of the fiber optic sensors. The strain flexibility estimated from the recorded data can be directly utilized, with no need to construct an analytical model. Here, the “space resolution” represents the region one sensor covers. Once the damage is localized, the number of unknown parameters to be identified can be sharply reduced in the damaged domain, in which the strain flexibility-based sensitivity function will be constructed for Level 2 damage quantification. This step-by-step procedure allows for damage quantification by reducing the significant number of unknown parameters in sensitivity equations.

### 3.2. Theoretical Basis of the Proposed Method

#### 3.2.1. Strain Flexibility Identification

The strain flexibility identified by long-gauge FBG sensors has been illustrated in previous research by the authors. In the work of [25], the identification method is briefly introduced as follows. When a force, $f_{p}$, is applied at node *p*, the corresponding long-gauge strain FRF of the *m*th element is as follows:

(1) $$\begin{array}{l} H_{mp}^{\bar{\varepsilon }}\left(\omega \right)=\frac{{\bar{\varepsilon }}_{m}\left(\omega \right)}{f_{p}\left(\omega \right)}=\frac{h_{m}}{L_{m}}\cdot \frac{\left(\theta_{i}\left(\omega \right)-\theta_{j}\left(\omega \right)\right)}{f_{p}\left(\omega \right)}\\ =\eta_{m}\cdot \left(H_{ip}^{d}\left(\omega \right)-H_{jp}^{d}\left(\omega \right)\right) \end{array},$$

where ${\bar{\varepsilon }}_{m}\left(\omega \right)$ and $f_{p}\left(\omega \right)$ are spectra of the long-gauge strain ${\bar{\varepsilon }}_{m}\left(t\right)$ and force $f_{p}\left(t\right)$, i and j are two nodes of the *m*th element, and $\theta_{i}$ and $\theta_{j}$ represent the rotational displacements of the *i* and *j* nodes. $H_{ip}^{d}\left(\omega \right)$ and $H_{jp}^{d}\left(\omega \right)$ are the displacement FRF in the rotation direction. $\eta_{m}=h_{m}/L_{m}$, $h_{m}$ is the distance from the element bottom surface to the beam neural axis, and $L_{m}$ is the element length.

A complex mode indicator function (CMIF) method is utilized to identify structural modal parameters. The singular value decomposition (SVD) technique is applied to Equation (1), and it is derived that

(2) $$H^{\bar{\varepsilon }}\left(\omega_{k}\right)=U^{\bar{\varepsilon }}S^{\bar{\varepsilon }}{\left(V^{\bar{\varepsilon }}\right)}^{T}=\eta_{m}\left(U_{L}S_{L}V_{L}^{T}-U_{R}S_{R}V_{R}^{T}\right),$$

where $U^{\bar{\varepsilon }}\in {ℜ}^{N_{0}\times N_{0}}$ is the left orthogonal matrix, which relates to the long-gauge strain mode shape; $V\in {ℜ}^{N_{i}\times N_{i}}$ is the right orthogonal matrix, $V_{L}$ and $V_{R}$ are the same because they consist of information about the displacement modal participation factors corresponding to the same impacting locations; $S\in {ℜ}^{N_{o}\times N_{i}}$ is the singular value matrix, and $S_{L}$ and $S_{R}$ are also the same because they consist of the information concerning frequency and damping ratio for the same structure. $U_{L}$ is the singular matrix including the information of the mode shapes related to the left nodes of all the elements, and $U_{R}$ is for the right node of all the elements. Thus, they are different. Equation (2) can be rewritten as

(3) $$H^{\bar{\varepsilon }}\left(\omega_{k}\right)=\eta_{m}\left(U_{L}-U_{R}\right)SV^{T}=U^{\bar{\varepsilon }}SV^{T}.$$

It is also known that the long-gauge strain FRF can be written in the following format:

(4) $$H^{\bar{\varepsilon }}\left(\omega_{k}\right)=\phi^{\bar{\varepsilon }}\left[\frac{1}{j\omega_{k}-\gamma_{r}}\right]L^{T},$$

where $\phi^{\bar{\varepsilon }}$ is the strain mode shape, $\left[\frac{1}{j\omega_{k}-\gamma_{r}}\right]=\left[\begin{array}{ccc} \frac{1}{j\omega_{k}-\gamma_{1}} & \cdots & 0\\ \vdots & \ddots & \vdots \\ 0 & \cdots & \frac{1}{j\omega_{k}-\gamma_{N}^{*}} \end{array}\right]$,$\gamma_{r}=-\xi_{r}\omega_{r}+j\omega_{r}\sqrt{1-\xi_{r}^{2}}$, and $\gamma_{r}^{*}$ is the conjugate of $\gamma_{r}$. $\omega_{r}$ is the structural frequency, $\xi_{r}$ is the damping ratio, and L is the modal participation matrix in which $L_{r}=Q_{r}\cdot \phi_{r,drv}$ for the *r*th mode, $\phi_{r,drv}$ is the mode shape vector of the driving point, and $Q_{r}$ is the modal scaling factor.

It can be seen that Equation (3) is the real mode form of the strain FRF while Equation (4) is the complex mode form. Basically, they are the same and, thus, the structural modal parameters including natural frequencies, damping ratios, and mode shapes can be identified from Equation (3). The modal scaling factor $Q_{r}$ can be solved from the least squares estimation formulation as follows:

(5) $$\frac{1}{Q_{r}}=C_{1r}C_{2r}{\left\{\begin{array}{c} eH^{\bar{\varepsilon }}{\left(\omega_{1}\right)}_{r}\\ eH^{\bar{\varepsilon }}{\left(\omega_{2}\right)}_{r}\\ \vdots \\ eH^{\bar{\varepsilon }}{\left(\omega_{k}\right)}_{r} \end{array}\right\}}^{+}\left\{\begin{array}{c} 1/\left(j\omega_{1}-\lambda_{r}\right)\\ 1/\left(j\omega_{2}-\lambda_{r}\right)\\ \vdots \\ 1/\left(j\omega_{k}-\lambda_{r}\right) \end{array}\right\},$$

where $C_{1r}={\left(U_{r}^{\bar{\varepsilon }}\right)}^{T}\phi_{r}^{\bar{\varepsilon }}$; $C_{2r}={\left(\phi_{r,drv}^{d}\right)}^{T}V_{r}$; and $eH^{\bar{\varepsilon }}{\left(\omega_{k}\right)}_{r}={\left(U_{r}^{\bar{\varepsilon }}\right)}^{T}\left[H^{\bar{\varepsilon }}\left(\omega_{k}\right)\right]V_{r}$, which is the enhanced FRF of the *r*th model. Then, based on the modal scaling factors and basic modal parameters, the structural long-gauge strain flexibility can be obtained in Equation (6):

(6) $$F^{\bar{\varepsilon }}\left(\omega \right)=\sum_{r=1}^{N}\left(\frac{Q_{r}\phi_{r}^{\bar{\varepsilon }}{\left(\phi_{r}^{d}\right)}^{T}}{-\gamma_{r}}+\frac{Q_{r}^{*}\phi_{r}^{\bar{\varepsilon }*}{\left(\phi_{r}^{d*}\right)}^{T}}{-\gamma_{r}^{*}}\right),$$

where $\phi_{r}^{\bar{\varepsilon }}$ is the long-gauge strain mode shapes identified from $U^{\bar{\varepsilon }}$; $\phi_{r}^{d}$ is the displacement mode shapes calculated from strain mode shapes by using the improved conjugate beam approach, and the symbol * represents the complex conjugate.

Equation (6) indicates that the estimation of the long-gauge strain flexibility does not require one to know the mass of the structure. It just requires the natural frequencies $\omega_{r}$, damping ratios $\xi_{r}$, identified strain modal shapes $\phi_{r}^{\bar{\varepsilon }}$, displacement mode shapes $\phi_{r}^{d}$, and modal scaling factors $Q_{r}$ to be known. For the *r*th mode, the relationship between the modal scaling factor and the modal mass can be derived as $Q_{r}=\frac{1}{2j\omega_{r}M_{r}}$. See Appendix A for more details. If $M_{r}=1$, the mass-normalized mode shape can be denoted as $\psi_{r}^{d}=\alpha_{r}\phi_{r}^{d}=\sqrt{2j\omega_{r}Q_{r}}\phi_{r}^{d}$. Replacing the unscaled mode shape in Equation (6) with the mass normalized mode shape $\psi_{r}^{d}$ and the corresponding strain mode shape $\psi_{r}^{\bar{\varepsilon }}$, the strain flexibility will be derived in another form:

(7) $$F^{\bar{\varepsilon }}\left(\omega \right)=\sum_{r=1}^{N}\frac{\psi_{r}^{\bar{\varepsilon }}{\left(\psi_{r}^{d}\right)}^{T}}{\omega_{r}^{2}}.$$

#### 3.2.2. Two-Level Corrosion Detection

Corrosion generally produces reduction of the stiffness of a structure, and these changes will lead to variation in strain flexibility. Thus, the difference between the strain flexibility for intact and corroded structures may be used to detect the corrosion damage in Level 1:

(8) $$\Delta F^{\bar{\varepsilon }}\left(\omega \right)=F_{1}^{\bar{\varepsilon }}\left(\omega \right)-F_{2}^{\bar{\varepsilon }}\left(\omega \right),$$

where $F_{1}^{\bar{\varepsilon }}\left(\omega \right),F_{2}^{\bar{\varepsilon }}\left(\omega \right)$ are the strain flexibility of the intact and the damaged structures, respectively. To amplify the difference for easy identification, a damage index is constructed by extracting the diagonal elements of the difference matrix:

(9) DI=10^(|diag(ΔFε¯)|max|diag(ΔFε¯)|)

In Level 2, the strain flexibility-based sensitivity equation is derived to quantify the damage. From Equation (7), the derivative of the strain flexibility with respect to the *i*th element stiffness is

(10) $$\frac{\partial F_{mq}^{\bar{\varepsilon }}}{\partial k_{i}}=\sum_{r=1}^{N}\left\{-\frac{2}{\omega_{r}^{3}}\frac{\partial \omega_{r}}{\partial k_{i}}\psi_{mr}^{\bar{\varepsilon }}\psi_{qr}^{d}+\frac{1}{\omega_{r}^{2}}\left(\frac{\partial \psi_{mr}^{\bar{\varepsilon }}}{\partial k_{i}}\psi_{qr}^{d}+\psi_{mr}^{\bar{\varepsilon }}\frac{\partial \psi_{qr}^{d}}{\partial k_{i}}\right)\right\}.$$

The equilibrium equation for the undamaged structural vibration equation is

(11) $$\left(-\omega_{r}^{2}\left[M\right]+\left[K\right]\right)\psi_{r}^{d}=\left\{0\right\}.$$

Applying the derivative to Equation (11) with respect to element stiffness $k_{i}$, it is derived that

(12) $$\left(-2\omega_{r}\frac{\partial \omega_{r}}{\partial k_{i}}\left[M\right]-\omega_{r}^{2}\frac{\partial \left[M\right]}{\partial k_{i}}+\frac{\partial \left[K\right]}{\partial k_{i}}\right)\psi_{r}^{d}+\left(-\omega_{r}^{2}\left[M\right]+\left[K\right]\right)\frac{\partial \psi_{r}^{d}}{\partial k_{i}}=0.$$

As the mass matrix [M] is independent of $k_{i}$, $\partial \left[M\right]/\partial k_{i}=0$. Multiply ${\left(\psi_{r}^{d}\right)}^{T}$ on both sides of this equation and the sensitivity coefficient of the *r*th natural frequency with respect to the *i*th element flexural stiffness $k_{i}$ can be derived:

(13) $$\frac{\partial \omega_{r}}{\partial k_{i}}=\frac{1}{2\omega_{r}}{\left(\psi_{r}^{d}\right)}^{T}\frac{\partial \left[K\right]}{\partial k_{i}}\psi_{r}^{d},\left(r,i=1,2,\ldots ,n\right).$$

Since the mode shapes of a structure are independent from each other and, thus, can form a complete mode shape space, the sensitivity coefficients of the *r*th mode shape can be expressed in the form of mode shape:

(14) $$\frac{\partial \psi_{r}^{d}}{\partial k_{i}}=\sum_{L=1}^{N}\alpha_{L}\psi_{L}^{d},$$

where $\alpha_{L}$ is the weight coefficient. Substitute Equation (14) into Equation (12) and multiply ${\left(\psi_{s}^{d}\right)}^{T}\left(s\neq r\right)$ on both sides. Since the mode shapes are orthogonal, two different modes will yield ${\left(\psi_{s}^{d}\right)}^{T}\left[M\right]\psi_{r}^{d}=0$ and ${\left(\psi_{s}^{d}\right)}^{T}\left[K\right]=\omega_{r}^{2}{\left(\psi_{s}^{d}\right)}^{T}\left[M\right]$. Then, Equation (12) can be rewritten as

(15) $${\left(\psi_{s}^{d}\right)}^{T}\frac{\partial \left[K\right]}{\partial k_{i}}\psi_{r}^{d}+\alpha_{s}\left(\omega_{s}^{2}-\omega_{r}^{2}\right)=0,$$

solving for $\alpha_{s}$:

(16) $$\alpha_{s}=\frac{1}{\omega_{r}^{2}-\omega_{s}^{2}}{\left(\psi_{s}^{d}\right)}^{T}\frac{\partial \left[K\right]}{\partial k_{i}}\psi_{r}^{d}\left(r\neq s\right).$$

When s=r, $\alpha_{s}=0$ can be derived.

The long-gauge strain mode shape $\psi_{mr}^{\bar{\varepsilon }}$ can be written as follows:

(17) $$\psi_{mr}^{\bar{\varepsilon }}=\frac{h_{m}}{L_{m}}\left(\psi_{or}^{d}-\psi_{pr}^{d}\right).$$

The derivative of the *r*th strain mode shape in terms of $k_{i}$ can be derived from the displacement mode shape sensitivity:

(18) $$\begin{array}{l} \frac{\partial \psi_{mr}^{\bar{\varepsilon }}}{\partial k_{i}}=\frac{h_{m}}{L_{m}}\left(\frac{\partial \psi_{or}^{d}}{\partial k_{i}}-\frac{\partial \psi_{pr}^{d}}{\partial k_{i}}\right)=\frac{h_{m}}{L_{m}}\left(\sum_{s=1}^{N}\alpha_{s}\psi_{os}^{d}-\sum_{s=1}^{N}\alpha_{s}\psi_{ps}^{d}\right)=\sum_{s=1}^{N}\alpha_{s}\left[\frac{h_{m}}{L_{m}}\left(\psi_{os}^{d}-\psi_{ps}^{d}\right)\right]\\ \;=\sum_{s=1}^{N}\alpha_{s}\psi_{ms}^{\bar{\varepsilon }} \end{array}$$

Substituting Equations (13), (14), (16), and (18) into Equation (10) can yield the sensitivity coefficients of the strain modal flexibility:

(19) $$\frac{\partial F_{mq}^{\bar{\varepsilon }}}{\partial k_{i}}=\sum_{r=1}^{N}\left\{-\frac{1}{\omega_{r}^{4}}{\left(\psi_{r}^{d}\right)}^{T}\frac{\partial \left[K\right]}{\partial k_{i}}\psi_{r}^{d}\psi_{mr}^{\bar{\varepsilon }}\psi_{qr}^{d}+\frac{1}{\omega_{r}^{2}}\left(\sum_{s=1}^{N}\alpha_{s}\psi_{ms}^{\bar{\varepsilon }}\right)\psi_{qr}^{d}+\frac{1}{\omega_{r}^{2}}\left(\sum_{s=1}^{N}\alpha_{s}\psi_{qs}^{d}\right)\psi_{mr}^{\bar{\varepsilon }}\right\}.$$

The strain flexibility can be expanded into a first-order Taylor’s series, as below:

(20) $$\Delta F_{mq}^{\bar{\varepsilon }}=\sum_{i=1}^{n}\frac{\partial F_{mq}^{\bar{\varepsilon }}}{\partial k_{i}}\cdot \Delta k_{i}.$$

The sensitivity function with respect to strain flexibility is obtained:

(21) $${\left[S\right]}_{M\times Nu}\cdot {\left\{\Delta k\right\}}_{Nu}={\left\{\Delta F_{}^{\bar{\varepsilon }}\right\}}_{M},S_{mq}=\frac{\partial F_{mq}^{\bar{\varepsilon }}}{\partial k_{i}},$$

where [S] is the sensitivity matrix obtained from the initial structural state. Δk is the change of the element stiffness, and $\Delta F_{}^{\bar{\varepsilon }}$ is the variation of strain flexibility. *Nu* is the number of parameters to be identified, and *M* is the number of columns for strain flexibility.

Before the corrosion damage is identified in Level 2, an original finite element (FE) model needs to be built to obtain the global stiffness matrix [K], from which the sensitivity matrix [S] can be determined. From Equation (21), it can be found that once the damage caused by corrosion is localized, the unknown parameters have been reduced from the entire structure to detected domain, upon which the quantification process can only focus. Thus, the convergence and reliability of the solution will achieve better results.

## 4. Numerical Example of a Steel Beam

**Figure 3:**
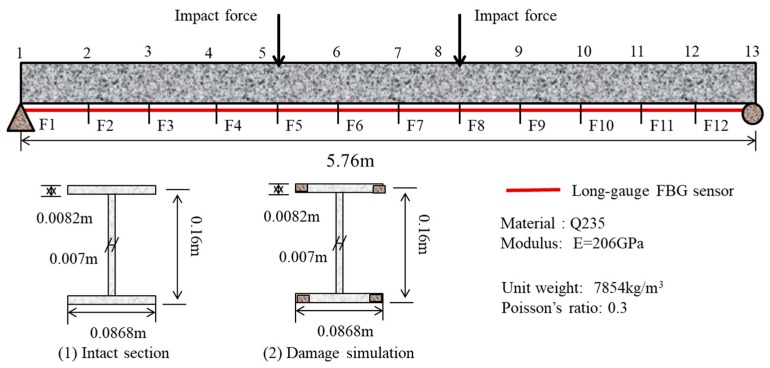
Configuration of the steel beam.

As shown in Figure 3, a simply supported steel beam model is used to study the validity of the proposed method. The beam has a length of 5.76 m, which is divided into 12 elements. The material is Q235 steel, with the elasticity of modulus 206 GPa, and the unit weight is 7854 kg/m^3^. Assume that 12 long-gauge FBG sensors with a gauge length of 0.48 m are mounted at the bottom of the beam to measure the macrostrain response of the structure. The numerical model is simulated in SAP2000 software and a Rayleigh damping matrix is adopted. Other than the baseline structure, three damage patterns are considered: (1) Case 1, single damage scenario for 5% stiffness loss at the fifth element; (2) Case 2, single damage scenario for 10% stiffness loss at the fifth element; (3) Case 3, multiple damage scenarios for 10% and 15% stiffness loss at the fifth and ninth elements. The corrosion damage is simulated by reducing the width of the flange. Impacting forces are applied on the fifth and eighth nodes as excitation. White noises (5%) are added into the response data to act as the observation noise.

**Figure 4:**
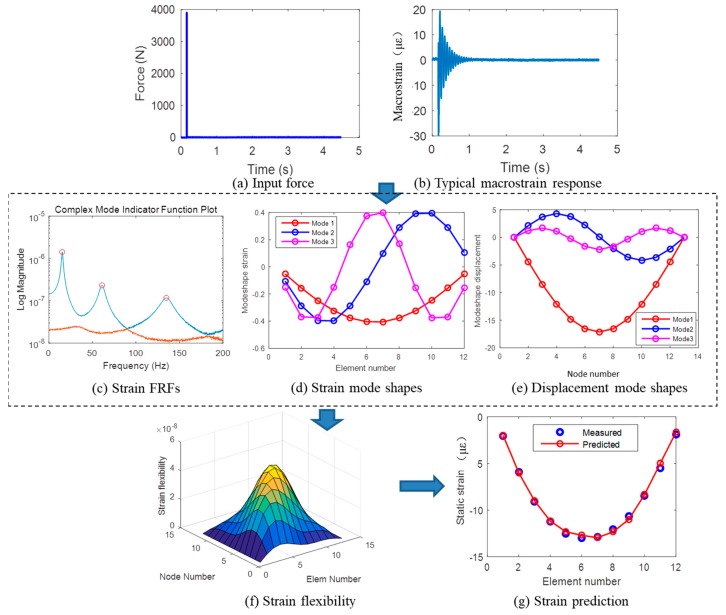
Process of strain flexibility identification for intact structure.

We take the intact structure as an example to illustrate the process of strain flexibility identification. By performing an impact test on the beam, the corresponding macrostrain responses are recorded. The applied impacting force and strain response are plotted in Figure 4a,b. Long-gauge strain FRFs are estimated from the macrostrain responses during the impact. According to Equation (3), the singular value decomposition is applied on the estimated strain FRF to obtain the singular matrix, which is plotted in Figure 4c. As shown in the figure, two curves are plotted due to the fact that two nodes were impacted during the test. Three peaks in the spectral line represent three modes that are identified. The corresponding macrostrain mode shapes and displacement mode shapes are plotted in Figure 4d,e. The natural frequencies in the first three modes are identified as 15.46, 61.2, and 135.37 Hz, and the corresponding damping ratios are identified as 0.5%, 1.1%, and 0.56%, respectively. According to Equation (5), the modal scaling factors can be obtained. Thus, based on the identified basic modal parameters, macrostrain mode shapes, and displacement mode shapes, the macrostrain flexibility is estimated from Equation (6) and plotted in Figure 4f. To verify the accuracy of the estimated strain flexibility, static strains measured from the corresponding static test are plotted for comparison (Figure 4g). The static test is performed by placing four static forces with a magnitude of 100 N each on nodes 3, 5, 8, and 10. It can be seen in each case that the predicted strain has good agreement with the measured strain.

**Figure 5:**
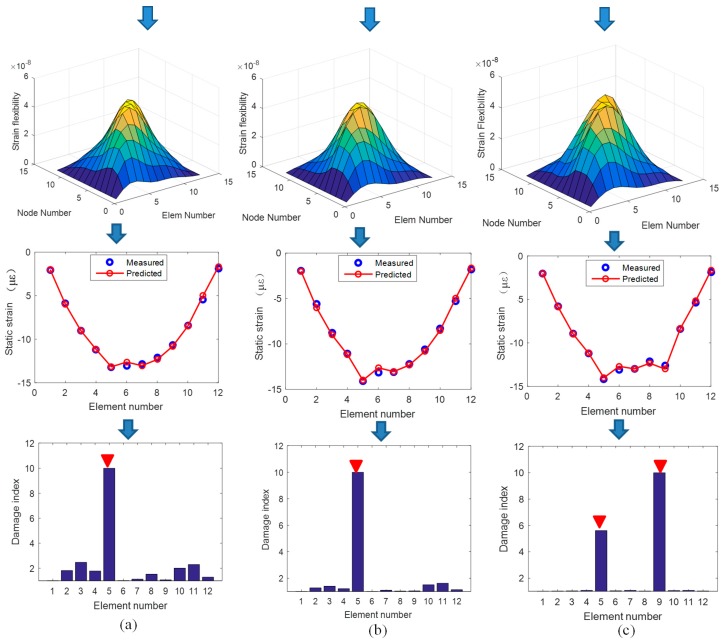
Damage location for Level 1. (**a**) Case 1; (**b**) Case 2; (**c**) Case 3.

Similar procedures are performed on corroded structures to obtain the corresponding structural strain flexibility, which has been plotted in Figure 5. Structural deformation predicted by the strain flexibility is also plotted to compare with the static test results provided by SAP2000. As shown in the figure, there is a good agreement between the predicted strain from the identified strain flexibility and the measured strain from the static test, demonstrating the accuracy of the strain flexibility identification. Based on the strain flexibility, the difference matrices are calculated, and the diagonal elements are extracted to locate the corrosion damage. The results are plotted in Figure 5a–c for three damage cases. From the figures, it can be found that the location of structural damage can be clearly detected. Therefore, strain flexibility difference is a good indicator for locating corrosion and is suitable for assessing singular and multiple incidents of damage.

**Figure 6:**
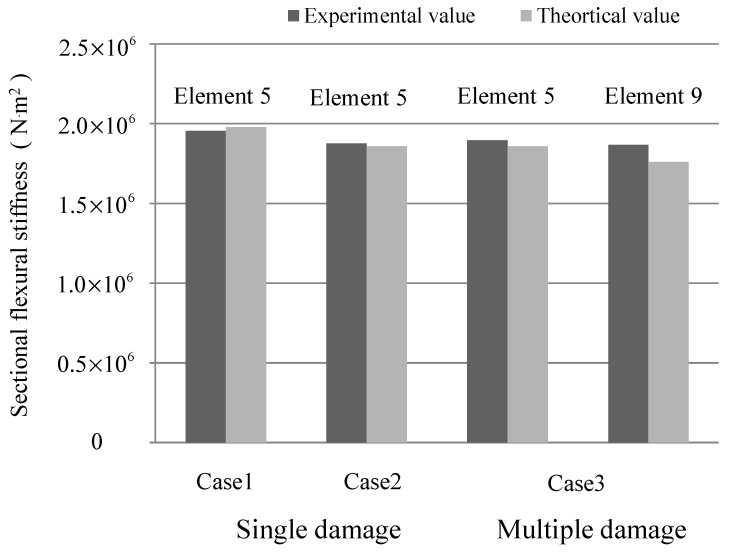
Damage quantification for three cases.

Once the corrosion damage is located, the corrosion quantification can be focused on the detected domain and, hence, the number of structural parameters to be identified is greatly reduced. Here, the sectional flexural rigidity of element 5 for cases 1 and 2, and sectional flexural rigidity of element 5 and 9 for case 3, are taken as the objective parameters for damage quantification. According to the scaling factor $Q_{r}$ identified from Equation (5), the coefficient $\alpha_{r}$ of the first three modes is calculated as 0.0003, 0.0012, and 0.0022. Thus, the mass normalized shape can be obtained, and the sensitivity coefficients of frequency, displacement mode, and strain mode that correspond to the detected element stiffness can be calculated from Equation (13), (14), and (18). Then, the stiffness reduction will be quantified from Equation (21), and the results are listed in Table 1 for comparison against the theoretical value. It can be found that the damage is quantified effectively using the strain flexibility-based sensitivity function, and the errors are all within an acceptable range. Figure 6 describes the result in a more intuitive way, and the identified value agrees with the theoretical value.

## 5. Experimental Verification through a Reinforced Concrete (RC) Beam

In Section 4, the applicability of the proposed method was demonstrated by a numerical example. This section is devoted to verifying the robustness of the long-gauge FBG sensors and the effectiveness of the proposed method by an experimental study of a simply supported reinforced concrete (RC) beam.

### 5.1. Description of the Experimental Setup

**Figure 7:**
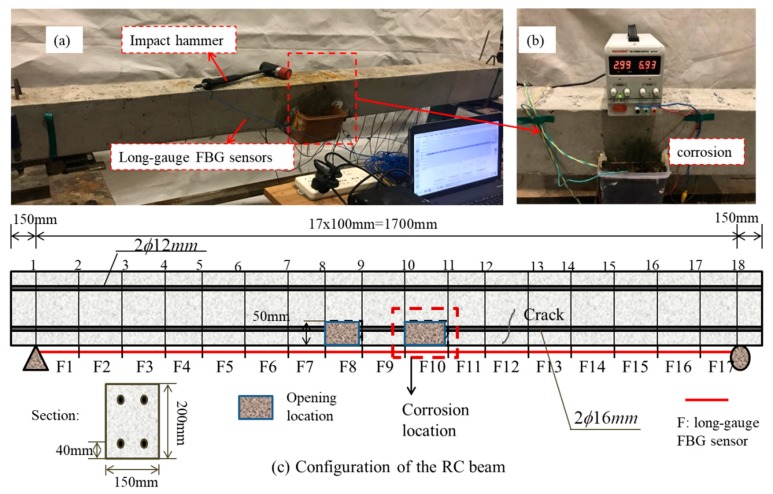
Illustration for the reinforced concrete (RC) beam; (**a**) The tested RC beam; (**b**) Corrosion setup; (**c**) Configuration of the RC beam.

The experimental beam has a total size of 2000 mm × 150 mm × 200 mm, and is reinforced with two deformed bars with a diameter of 16 mm and two compression bars with a diameter of 12 mm. The configuration is illustrated in Figure 7. It was been divided into 17 elements, and a visible crack runs through the twelfth element, as shown in Figure 7c.

Corrosion is conducted via the accelerated corrosion technique. Seventeen long-gauge FBG sensors with a gauge length of 100 mm were mounted on the bottom of the beam surface, as shown in Figure 7. The long-gauge FBG sensors were fixed using reinforcement glue on the designed locations. All sensors were pre-tensioned during the fabrication to ensure the accurate signals of low-level strains. Before installing the long-gauge FBG sensors, the concrete surface was firstly cleaned by using a sander to remove the cement cover, then alcohol was applied to scrub the concrete surface to make sure that the FBG sensors can be fixed on the concrete surface sustainably by using epoxy primer.

### 5.2. Accelerated Corrosion Procedure

#### 5.2.1. Calibration Test

**Figure 8:**
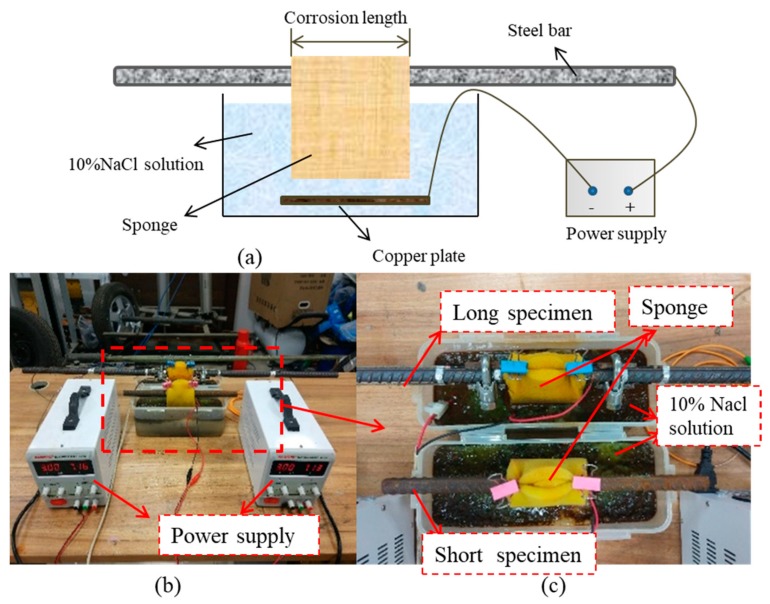
Corrosion calibration test; (**a**) Illustration of the accelerated corrosion; (**b**) Calibration test; (**c**) Details of two specimen.

To accelerate the corrosion process, the electrochemical corrosion method was applied to a steel bar similar to those used for reinforcing the RC beam. A corrosion calibration test was conducted to determine the theoretical corrosion amount in the specified time. The setup is illustrated in Figure 8a. It can be seen from the figure that the steel bar was immersed in 10% sodium chloride (NaCl) solution to create a path for the current between the anode and cathode terminals. A power supply was set to create a stable 3 A current output. To eliminate the effects of sample length on corrosion, two samples with different lengths (325 and 1000 mm) were tested (Figure 8b–c). However, the length of the corroded part is 78 mm for both samples.

According to the Faraday’s first law of electrolysis, the weight loss of steel within a specified time can be calculated as follows:

(22) $$\Delta W=\frac{\omega \times i\times t}{F},$$

where ΔW is the weight loss of the steel, ω=M/n is the constant, *n* is the number of electrons exchanged during the corrosion process (*n* = 2 for Fe^2+^), and M is the molar mass of iron (i.e., M = 5584 g/mol). i is the applied current in A, t is the corrosion time, and F is Faraday’s constant (96,487 C/mol).

It can be concluded from Equation (22) that the corrosion of the steel bar depends only on the current intensity i. Therefore, the relationship between corrosion weightless rates and time derived from calibration tests is similar to the corrosion that occurs in the RC beam if the electrochemical corrosion can be controlled under the same conditions. To ensure the sustainability of the corrosion progress, the corrosion solution was changed every two hours, the rust adhering to the corroded section was carefully cleaned, then the quality of the remaining bar was measured to obtain the weight loss that occurred during corrosion. The corrosion process lasted for 8 h, and the residual mass of each sample was measured every 2 h; the values are recorded in Table 2. The varying rate of the steel section area can be calculated as follows:

(23) $$r=\frac{\Delta A}{A}=\frac{\Delta W}{W},$$

where ΔA, ΔW are the loss in area and mass for corroded part; and A, W are the area and mass for the corroded part in the initial state. For long specimens, W=78/1000×1517.07=118.33 g; for short specimens, W=78/325×454.5=109.08 g.

The relationships of the average weight loss ratio with time are described in Table 2. It can be seen that the corrosion level increases linearly with time and the whole length of the steel bar has no effect on the corrosion rate. Therefore, the calibration result can be considered as the theoretical value. In addition, this test confirmed that certain packaging can effectively protect the FBG sensors from electric effects, long-term salt attacks, and temperature variations experienced during the corrosion process. As the Young’s modulus of concrete and steel bars is needed to quantify the damage, cube compression and steel tensile tests were conducted to determine the modulus.

#### 5.2.2. Corrosion Setup

To make the beam easier to corrode, two small openings with a size of 100 mm × 30 mm × 50 mm are introduced on the bottom surface of the RC beam, and corrosion was applied to one of the main reinforcements through the openings.

The corrosion was introduced on element 10. A plastic bottle with a height of 80 mm, as shown in Figure 7b, was fixed to the RC beam surface with epoxy resin. Then, 10% sodium chloride (NaCl) solution was poured into the plastic bottle to a height of 60 mm to guarantee that the exposed deformed bar was immersed in the solution. The corrosion solution was changed every two hours to ensure the sustainability of the corrosion progress. Two corrosion cases were considered. The first case lasted for 8 h, and the second case lasted for 16 h. They are defined as case 1 and case 2 in the following section.

### 5.3. Two Level Strategy for Corrosion Damage Quantification

#### 5.3.1. Level 1: Corrosion Damage Localization

Impact testing was performed on the RC beam to obtain the macrostrain flexibility. The impacting force was applied on the nodes from left to right by using the PCB model 086D20 short-sledge impulse hammer and then the corresponding macrostrain responses were measured by the SM130 optical sensing interrogator. The NI PXIe-1082 data acquisition system was used for impacting force measurements and the sampling frequencies are both set as 1000 Hz. The impacting force and macrostrain response were recorded to estimate the strain FRF, and then the basic modal parameters were identified using the CMIF method. For the intact structure, the first natural frequency identified from the impact test data was 56.58 Hz, and the modal scaling factor was $1.45\times 10^{-5}$. The strain mode shapes were identified from Equation (3) and then the improved conjugated beam method was applied to obtain the corresponding displacement mode shapes. As the higher mode frequency of the RC beam was large, its contribution to the flexibility may be neglected. The exact strain flexibility may be obtained by considering only the first mode.

**Figure 9:**
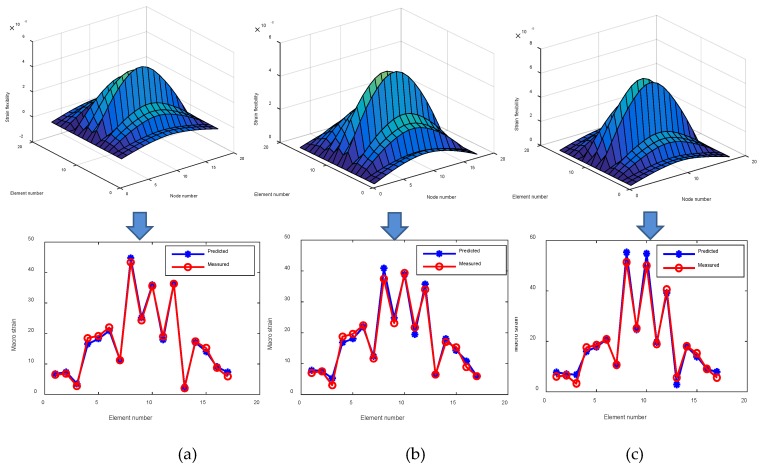
Strain flexibility and static strain prediction for intact structures. (**a**) Intact structure; (**b**) Case 1; (**c**) Case 2.

After the basic modal parameters and modal scaling factors are identified in each condition, the structural strain flexibility was estimated. Figure 9 plots the strain flexibility for the intact and corroded structures for case 1 and case 2. A static test was also performed by placing two mass blocks with weights of 61.2 kg and 61.3 kg, respectively, on node 6 and node 13, to demonstrate the accuracy of the strain flexibility calculated from the impact test. For the intact and corroded structures, the identified strain from the flexibility and measured static strain are plotted in Figure 9 for comparison. Owing to the presence of an opening, the strain of elements 8 and 10 increased sharply. Since a crack existed in element 12, the strain was also very large. As shown in the figure, the predicted strain from the flexibility is in accordance with the measured strain from the long-gauge FBG sensors, which demonstrates the accuracy of the estimated strain flexibility. To eliminate the effect caused by temperature, impact and static tests for each condition were all performed at the same time of day.

#### 5.3.2. Level 2: Corrosion Damage Quantification

**Figure 10:**
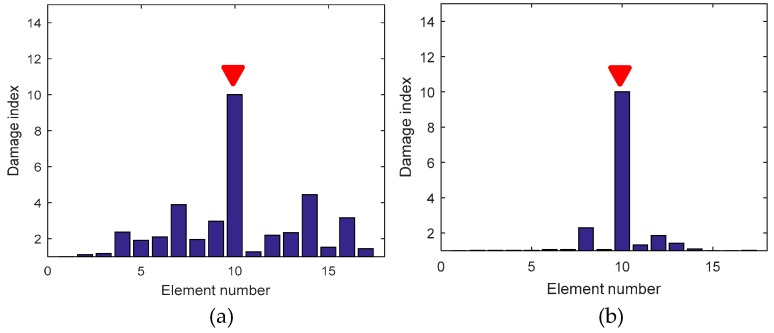
Corrosion detection for Level 1. (**a**) Damage index for Case 1; (**b**) Damage index for Case 2.

After the strain flexibility for each condition was obtained, the damage index was calculated by Equation (8) and plotted in Figure 10. In case 1, after 8 h of corrosion, the cross-sectional area of the steel bar was reduced by 22% according to the calibration test. As the contribution of the steel bar to the stiffness of the entire section was very small, a 22% steel area reduction only caused a 7% decrease in cross-sectional stiffness. Although the damage was small, the index located the damage successfully, which demonstrates the robustness of the FBG sensors for perceiving minor damage. In case 2, according to Faraday’s law, an area reduction of about 45% occurred on element 10. As the corrosion degree increased, the damage became easier to locate.

**Figure 11:**
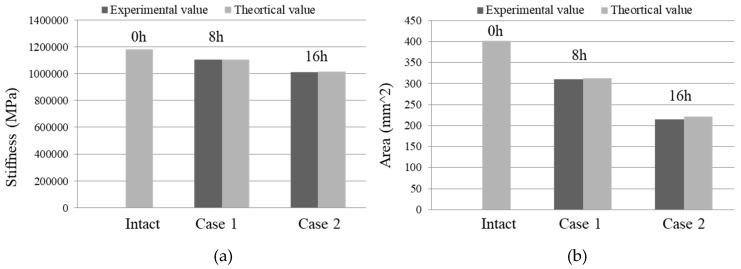
Corrosion quantification for Level 2. (**a**) Identified stiffness; (**b**) Identified steel area.

Once the damage location was detected in Level 1, the damage severity in terms of stiffness reduction for cross-sections could be quantified according to the Equation (21). The theoretical value for the structural sectional stiffness of the rectangular section under a short-term load can be calculated as follows [26]:

(24) $$B_{s}=\frac{E_{s}A_{s}h_{0}^{2}}{1.15\psi +0.2+6\alpha_{E}\rho },$$

where $\alpha_{E}=E_{s}/E_{c},\;\rho =A_{s}/bh_{0}$; $B_{s}$ is the sectional flexural rigidity; and $E_{s}$ and $A_{s}$ are the elastic modulus and section areas of the reinforcement, respectively. $E_{c}$ is the elastic modulus for concrete. $h_{0}$ is the effective height of the reinforced concrete section, and $\alpha_{E}=E_{s}/E_{c}$ is the elastic modulus ratio. ψ is the non-uniformity coefficient of strain subjected to longitudinal tension. Comparison between the theoretical value and the experimental results of the stiffness and steel areas are plotted in Figure 11a,b. It can be found that the experimental values agree with the theoretical values.

## 6. Conclusions

The overall conclusions of this research are that the distributed long-gauge FBG sensor is a potentially promising method for effective and accurate corrosion damage detection and further structural long-term performance evaluation.

(1) Based on the long-gauge FBG strain sensors, a new kind of corrosion detection methodology via impact test is proposed and demonstrated.
(2) The original contribution of this paper is the development of a step-by-step strategy that helps to locate and quantify corrosion damage by using a long-gauge FBG sensor; a solid theoretical basis has been developed to guarantee that this sensor will detect corrosion accurately.
(3) The proposed two-level corrosion detection methodology presents a distinct advantage in that locating Level 1 damage significantly reduces the number of unknown parameters in the sensitivity equations and increases the success of Level 2 corrosion quantification.

Both numerical and experimental examples have been conducted, and they verify the robustness of the novel long-gauge FBG sensors, as well as of the effectiveness of the proposed method for corrosion detection of in-service structures. The proposed two-level corrosion detection strategy that employs distributed long-gauge FBG sensors has a great application potential in civil infrastructural maintenance. In addition, an assumption that the steel of the damaged structure is still in linear elastic state is needed due to the fact that method of the strain flexibility identification is studied based on the theory of elasticity. Taking steel plasticity into consideration is worthy of further study in future works.

## 7. Patents

The proposed technique for corrosion detection is patent pending.

## Figures and Tables

**Table 1:** Damage quantification results.

Damage Location	Element 5			Element 9		
Damage Quantification	Theoretical Value ($N\cdot m^{2}$)	Experimental Value ($N\cdot m^{2}$)	Error (%)	Theoretical Value ($N\cdot m^{2}$)	Experimental Value ($N\cdot m^{2}$)	Error (%)
Case 1	$1.978\times 10^{6}$	$1.955\times 10^{6}$	1.16	-	-	-
Case 2	$1.858\times 10^{6}$	$1.876\times 10^{6}$	0.97	-	-	-
Case 3	$1.858\times 10^{6}$	$1.894\times 10^{6}$	1.93	$1.759\times 10^{6}$	$1.868\times 10^{6}$	6.2

**Table 2:** Corrosion calibration result with 3 A current.

	Short Specimen (325 mm)			Long Specimen (1000 mm)			
Time (h)	Residual Mass (g)	Lost Mass (g)	Weight Loss Ratio (%)	Residual Mass (g)	Lost Mass (g)	Weight Loss Ratio (%)	Mean of the Ratio (%)
0	454.5	0	0	1517.07	0	0	0
2	448	6.5	5.96	1510.65	6.42	5.44	5.7
4	441.5	13	11.92	1504.37	12.7	10.76	11.34
6	435	19.5	17.88	1497.89	19.18	16.25	17.1
8	429	25.5	23.38	1491.55	25.52	21.62	22.5

## Appendix A. Relationship between Modal Mass and Modal Scaling Factor

According to the complex modal theory, the long-gauge strain FRFs can be expanded as

(A1) $$H^{\bar{\varepsilon }}\left(\omega \right)=\sum_{r=1}^{N}\left(\frac{Q_{r}\phi_{r}^{\bar{\varepsilon }}{\left(\phi_{r}^{d}\right)}^{T}}{j\omega -\gamma_{r}}+\frac{Q_{r}^{*}\phi_{r}^{\bar{\varepsilon }*}{\left(\phi_{r}^{d*}\right)}^{T}}{j\omega -\gamma_{r}^{*}}\right),$$

where $Q_{r}$ is the *r*th scaling factor, $\phi_{r}^{\bar{\varepsilon }}$ and $\phi_{r}^{d}$ are the *r*th complex strain mode shape and displacement mode shape, and * denotes the conjugate.

According to real modal theory, the long-gauge strain FRFs is rewritten as below:

(A2) $$H^{\bar{\varepsilon }}\left(\omega \right)=\sum_{r=1}^{N}\frac{\varphi_{r}^{\bar{\varepsilon }}{\left(\varphi_{r}^{d}\right)}^{T}/M_{r}}{{\left(j\omega \right)}^{2}+\left(j\omega \right)2\xi_{r}\omega_{r}+\omega_{r}^{2}},$$

where $M_{r}$ is the *r*th modal mass, $\varphi_{r}^{\bar{\varepsilon }}$ and $\varphi_{r}^{d}$ are the *r*th real strain mode shape and displacement mode shape.

There is no essential difference between Equation (A1) and Equation (A2) as they are two different forms of long-gauge strain FRFs, thus, the following equation can be obtained:

(A3) $$H^{\bar{\varepsilon }}\left(\omega \right)=\sum_{r=1}^{N}\left(\frac{Q_{r}\phi_{r}^{\bar{\varepsilon }}{\left(\phi_{r}^{d}\right)}^{T}}{j\omega -\gamma_{r}}+\frac{Q_{r}^{*}\phi_{r}^{\bar{\varepsilon }*}{\left(\phi_{r}^{d*}\right)}^{T}}{j\omega -\gamma_{r}^{*}}\right)=\sum_{r=1}^{N}\frac{\varphi_{r}^{\bar{\varepsilon }}{\left(\varphi_{r}^{d}\right)}^{T}/M_{r}}{{\left(j\omega \right)}^{2}+\left(j\omega \right)2\xi_{r}\omega_{r}+\omega_{r}^{2}}.$$

For the *r*th mode, the same contribution is derived from the two representations:

(A4) $$\frac{Q_{r}\phi_{r}^{\bar{\varepsilon }}{\left(\phi_{r}^{d}\right)}^{T}}{j\omega -\gamma_{r}}+\frac{Q_{r}^{*}\phi_{r}^{\bar{\varepsilon }*}{\left(\phi_{r}^{d*}\right)}^{T}}{j\omega -\gamma_{r}^{*}}=\frac{\varphi_{r}^{\bar{\varepsilon }}{\left(\varphi_{r}^{d}\right)}^{T}/M_{r}}{{\left(j\omega \right)}^{2}+\left(j\omega \right)2\xi_{r}\omega_{r}+\omega_{r}^{2}}.$$

The complex mode shape can be normalized as real mode shape when the structural damping matrix can be orthogonalized, and it is derived that

(A5) $$\phi_{r}^{d}=\phi_{r}^{d*}=\varphi_{r}^{d},$$

(A6) $$\phi_{r}^{\bar{\varepsilon }}=\phi_{r}^{\bar{\varepsilon }*}=\varphi_{r}^{\bar{\varepsilon }}.$$

Substituting Equation (A5) and (A6) into Equation (A4), the following equation is derived:

(A7) $$\frac{Q_{r}\phi_{r}^{\bar{\varepsilon }}{\left(\phi_{r}^{d}\right)}^{T}}{j\omega -\gamma_{r}}+\frac{Q_{r}^{*}\phi_{r}^{\bar{\varepsilon }}{\left(\phi_{r}^{d}\right)}^{T}}{j\omega -\gamma_{r}^{*}}=\frac{\phi_{r}^{\bar{\varepsilon }}{\left(\phi_{r}^{d}\right)}^{T}/M_{r}}{{\left(j\omega \right)}^{2}+\left(j\omega \right)2\xi_{r}\omega_{r}+\omega_{r}^{2}},$$

where $\phi_{r}^{\bar{\varepsilon }}{\left(\phi_{r}^{d}\right)}^{T}$ is a matrix and the coefficients must be equal if the matrices on both sides of the above equation are equal, thus, it is derived at any frequency ω:

(A8) $$\frac{Q_{r}}{j\omega -\gamma_{r}}+\frac{Q_{r}^{*}}{j\omega -\gamma_{r}^{*}}=\frac{1/M_{r}}{{\left(j\omega \right)}^{2}+\left(j\omega \right)2\xi_{r}\omega_{r}+\omega_{r}^{2}},$$

when ω=0 and $\omega =\omega_{r}$, the following equations can be obtained:

(A9) $$\{\begin{array}{c} \frac{Q_{r}}{-\lambda_{r}}+\frac{Q_{r}^{*}}{-\lambda_{r}^{*}}=\frac{1/M_{r}}{\omega_{r}^{2}}\\ \frac{Q_{r}}{j\omega_{r}-\lambda_{r}}+\frac{Q_{r}^{*}}{j\omega_{r}-\lambda_{r}^{*}}=\frac{1/M_{r}}{{\left(j\omega_{r}\right)}^{2}+\left(j\omega_{r}\right)2\xi_{r}\omega_{r}+\omega_{r}^{2}} \end{array}.$$

The scaling factors $Q_{r}$ and $Q_{r}^{*}$ can be solved as

(A10) $$\{\begin{array}{c} Q_{r}=\frac{1}{j2M_{r}\omega_{r}\sqrt{1-\xi_{r}^{2}}}\\ Q_{r}^{*}=\frac{1}{-j2M_{r}\omega_{r}\sqrt{1-\xi_{r}^{2}}} \end{array}.$$

Usually, the damping ratio for civil structure is small so that $\sqrt{1-\xi_{r}^{2}}\approx 1$. Therefore, the relationship between modal mass and scaling factor can be derived as

(A11) $$\{\begin{array}{c} Q_{r}=\frac{1}{j2M_{r}\omega_{r}}\\ Q_{r}^{*}=\frac{1}{-j2M_{r}\omega_{r}} \end{array}.$$
