# Erratum: Cheng, Y.; Zhao, C.; Zhang, J.; Wu, Z. Application of a Novel Long-Gauge Fiber Bragg Grating Sensor for Corrosion Detection via a Two-Level Strategy. *Sensors* 2019, *19*, 954

**DOI:** 10.3390/s19153302

**Published:** 2019-07-26

**Authors:** Yuyao Cheng, Chenyang Zhao, Jian Zhang, Zhishen Wu

**Affiliations:** 1 School of Civil Engineering, Southeast University, Nanjing 210096, China; 2 Jiangsu Key Laboratory of Engineering Mechanics, Southeast University, Nanjing 210096, China; 3 International Institute for Urban System Engineering, Southeast University, Nanjing 210096, China

The authors wish to make the following erratum to this paper [1]. 

In the paper, the second author’s name Chengyang Zhao should be Chenyang Zhao and his e-mail, 220171151@seu.edu.cn, has been changed to cyzhao@seu.edu.cn.

The errors were introduced as a result of author oversight. We apologize for any inconvenience caused to the readers by these mistakes. The manuscript will be updated, and the original will remain online on the article website.
